# Esophageal atresia with tracheo-esophageal fistula: Role of nebulized N-acetylcysteine in the outcome

**DOI:** 10.4103/0971-9261.59612

**Published:** 2009

**Authors:** A. Pandey, A. N. Gangopadhyay, S. P. Sharma, V. Kumar

**Affiliations:** Department of Pediatric Surgery, Institute of Medical Sciences, Banaras Hindu University, Varanasi, 221005, U.P., India

Sir,

We would like to inform the readers about the beneficial effect of N-acetylcysteine (NAC) in the nebulization form for patients of esophageal atresia with tracheo-esophageal fistula (EA-TEF). In developing countries such as India, most of the babies of EA-TEF present late.[1] These patients have pneumonitis due to reflux of gastric content through the fistula and also due to aspiration of saliva. It results in the production of thick secretions in the respiratory tract, which may lead to blockage of the respiratory passage and may adversely affect the outcome of these patients.

NAC is a sulfhydryl-containing compound.[2] Its effectiveness as a mucolytic agent results from its sulfhydryl group interacting with disulfide bonds in mucoprotein with the mucus and subsequently being broken into smaller, less viscous units.[2] NAC may also act as an expectorant by stimulating both ciliary action and the gastro-pulmonary vagal reflex, thereby clearing the mucus from the airways. We, therefore, used it as a mucolytic agent in patients of EA-TEF.

After taking the written and informed consent from the attendants of the seven patients of EA-TEF, NAC was given in a nebulized form (1:5 dilution, every 4 hourly) both pre- and postoperatively. After its administration, the suction of liquefied respiratory secretions was easy. All these patients were operated upon in a day or two after admission instead the usual time of 2 to 3 days. The postoperative course was smooth for all, except one patient who expired.

This observation may show the beneficial effect of NAC in patients of EA-TEF. We do agree that this is not a randomized control trial to give any definite conclusion, but it does suggest that a planned study is needed to substantiate our observation. The idea of giving NAC in nebulized form is that the solution will be reaching to the site of action directly.

NAC has been used for a wide range of conditions such as antioxidant, hepatoprotectant, as mucolytic in various respiratory conditions,[2,3] but its use in EA-TEF in nebulized form has not been reported previously.

It appears that nebulized NAC may improve the outcome of the patients of EA-TEF. This observation, however, needs further documentation by way of prospective studies.
